# Radiation Pneumonitis Risk Assessment Using Fractal Analyses in NSCLC Patients Treated with Curative-Intent Radiotherapy

**DOI:** 10.3390/life15101596

**Published:** 2025-10-13

**Authors:** Jeongeun Hwang, Sun Myung Kim, Joon-Young Moon, Bona Lee, Jeongmin Song, Sookyung Lee, Hakyoung Kim

**Affiliations:** 1 Department of Medical IT Engineering, Soonchunhyang University, Asan-si 31538, Chungcheongnam-do, Republic of Korea; hwangje02@sch.ac.kr (J.H.); 2bona03@sch.ac.kr (B.L.); jmluv118@sch.ac.kr (J.S.); sooruxd@sch.ac.kr (S.L.); 2 Departments of Radiation Oncology, Korea University Guro Hospital, Korea University College of Medicine, Seoul 08308, Republic of Korea; sunmyung01@hanmail.net; 3 Center for Neuroscience Imaging Research, Institute for Basic Science, Suwon-si 16419, Republic of Korea; joon.young.moon@gmail.com; 4 Sungkyunkwan University, Suwon-si 16419, Republic of Korea

**Keywords:** non-small cell lung cancer, radiotherapy, radiation pneumonitis, fractal, imaging biomarker

## Abstract

**Objectives:** This study evaluated the utility of complex morphometric analyses for predicting radiation pneumonitis (RP) and proposed a quantitative prognostic framework for patients with non-small cell lung cancer (NSCLC) undergoing curative-intent radiotherapy (RT). Imaging biomarkers, including box-counting fractal dimension (BoxFD), lacunarity, and minimum spanning tree fractal dimension (MSTFD), were assessed for their prognostic significance. **Materials and Methods:** We retrospectively analyzed 166 NSCLC patients who received curative-intent RT and had both pre-treatment and follow-up chest CT scans. Among them, 85 received RT alone and 81 underwent concurrent chemoradiotherapy (CCRT). Fractal features were measured to build a Random Forest model (RFM) predicting RP of grade ≥ 2, and the most important features were used to construct a decision tree model. **Results:** RP of grade ≥ 2 occurred in 19 patients (22.3%) in the RT alone group and 44 patients (54.3%) in the CCRT group. Lacunarity increased significantly post-RT in both groups, while BoxFD and MSTFD showed no significant changes. In the RFM, pre-RT MSTFD and lung dose parameters (V10 in RT alone; V5–V20 in CCRT) were identified as key predictors. Decision tree models based on these features achieved high predictive performance, with AUROC of 0.83 and 0.85, and F1 scores of 0.92 and 0.76 for RT alone and CCRT groups, respectively. **Conclusions:** Fractal imaging biomarkers demonstrated promising prognostic value for predicting grade ≥ 2 RP in NSCLC patients. The proposed decision tree model may serve as a practical tool for early identification of high-risk patients, facilitating personalized treatment strategies and informing future research.

## 1. Introduction

Radiation pneumonitis (RP) is an inflammatory lung injury that typically develops within 3 to 6 months following thoracic radiotherapy and represents one of the most clinically significant complications in patients undergoing treatment for lung cancer. The clinical presentation of RP ranges from mild symptoms, such as cough and low-grade dyspnea, to severe respiratory distress requiring hospitalization and systemic corticosteroid therapy. In some cases, RP can independently affect morbidity and mortality and may lead to permanent lung scarring, known as pulmonary fibrosis. Pulmonary toxicity occurs in approximately 5–25% of patients receiving conventionally fractionated radiotherapy [1,2,3,4,5,6,7,8].

Several studies have investigated clinical, dosimetric, and treatment-related risk factors for RP in non-small cell lung cancer (NSCLC). For example, Claude et al. [2] conducted a prospective analysis of clinical and dosimetric parameters, Shi et al. [8] examined severe acute RP in patients with locally advanced NSCLC treated with concurrent chemoradiotherapy (CCRT) and intensity-modulated radiotherapy (IMRT), and Baker et al. [1] evaluated clinical and dosimetric predictors in patients undergoing stereotactic ablative radiotherapy (SABR). However, the precise risk factors for RP remain uncertain, and consistent predictors have yet to be established.

In previous studies, the authors have reported differences in the incidence of severe RP according to the presence and type of underlying lung disease [9,10,11]. In particular, idiopathic pulmonary fibrosis (IPF) is associated with a higher incidence of severe RP and can lead to rapid deterioration and poor prognosis. These observations highlight the need for improved risk stratification and the development of predictive models to guide personalized management of patients at high risk for treatment-related pulmonary complications [12,13,14,15].

Imaging features derived from computed tomography (CT) scans, including changes in lung density and texture, have been proposed as potential predictive markers for RP; however, their clinical application remains limited. Motivated by this gap, we aimed to investigate whether quantitative imaging biomarkers could serve as predictive indicators for RP, in combination with established clinical and dosimetric risk factors. Our initial analysis focused on a high-risk subgroup of patients with IPF, as RP in these individuals may directly affect survival outcomes. Subsequently, the analysis was expanded to the entire cohort of patients receiving thoracic radiotherapy during the same period.

In previous work, we demonstrated that morphometric complexity analyses—such as box-counting fractal dimension (BoxFD), lacunarity, and minimum spanning tree fractal dimension (MSTFD)—may provide potential imaging biomarkers for RP prognosis [16,17]. These complexity metrics quantify morphometric aspects of lung tissue connectivity, space-filling properties, and rotational invariance, offering insights into lung tissue integrity and potential susceptibility to radiation-induced injury.

However, our previous study was limited by its exclusive reliance on simulation CT scans, constraining the study design to a cross-sectional analysis. In the current study, we sought to augment the prognostic accuracy for RP by incorporating diagnostic chest CT imaging and evaluating changes in complexity biomarkers derived from longitudinal scans obtained both pre- and post-radiotherapy.

Finally, this study aimed to utilize complex morphometry analyses methods to assess the risks of RP and to propose a quantitative prognostic framework for patients with NSCLC undergoing curative-intent radiotherapy. The novelty of the study is that it is the first study to investigate the differences in morphometric complexity features before and after radiotherapy and to explore their prognostic potential in NSCLC patients undergoing radiotherapy.

## 2. Materials and Methods

### 2.1. Patients and Data Collection

This retrospective study analyzed the medical records of 166 patients diagnosed with NSCLC who received curative-intent radiotherapy and had both a pre-treatment diagnostic chest CT scan and a follow-up CT scan within 6 months after completing radiotherapy at a single institution between June 2019 and December 2022. Among these patients, 85 with stage I–II NSCLC were treated with definitive radiotherapy alone (RT alone), whereas 81 with stage III NSCLC received CCRT.

In this study, we specifically included only those patients who had undergone diagnostic chest CT within six months after the completion of radiotherapy. This criterion was applied because follow-up CT scans were essential for assessing early parenchymal changes in the lung and for correlating these findings with the onset of RP.

All patient data were de-identified to ensure patient privacy and comply with ethical guidelines. This study was approved by the Korea University Medical Center Institutional Review Board (no. 2023GR0216), and the requirement for consent to participate was waived by the Institutional Review Board review committee because of the retrospective nature of the study.

### 2.2. Treatment Scheme and Surveillance

In accordance with institutional protocols, patients with stage I–II NSCLC presenting with small-sized (≤4 cm) and peripherally located tumors were treated with SABR, delivered at a total dose of 60 Gy in four fractions. Patients who did not meet these criteria were treated with IMRT, receiving a total dose of 60 Gy in 20 fractions. Patients with stage III NSCLC were treated using IMRT with a simultaneous integrated boost (SIB) technique, delivering a total dose of 66/60 Gy in 30 fractions.

Treatment-related toxicities were assessed using the Common Terminology Criteria for Adverse Events (CTCAE), version 5.0. In this study, radiation pneumonitis (RP) of grade 2 was characterized by the need for corticosteroid administration in an out-patient setting.

### 2.3. CT Scanning and Morphometric Complexity Analyses

The patients were scanned by Aquilion Lightning 80 (Cannon Medical Systems, Otawara, Japan), without contrast agent, and reconstructed to 1.0 × 1.0 × 2.5 mm voxel spacing DICOM format with a soft kernel. Pre-RT and post-RT CT scans were retrieved and processed for morphometric complexity analyses. The lung parenchyma was segmented from the CT images manually and binary masks for normal attenuation areas (NAAs) were defined at >−950 HU and ≤−700 HU. Three distinct morphometric complexity analysis methods were employed: BoxFD, lacunarity, and MSTFD.

BoxFD was calculated by covering the segmented lung region with boxes of varying sizes and counting the number of boxes required to cover the structure. The BoxFD reflects the complexity and irregularity of the lung tissue. A higher BoxFD indicates a more complex and irregular structure.

Lacunarity quantifies the translational and rotational invariance of a fractal pattern, providing information about the distribution of gaps or “lacunae” within the structure. It complements BoxFD by describing the texture and heterogeneity of the lung parenchyma. A higher lacunarity value suggests a more gappy or clumpy distribution.

MSTFD was derived from the minimum spanning tree constructed on the segmented lung region, where nodes represent voxels and edges represent distances between them. MSTFD provides insights into fiber network connectivity.

All fractal dimension calculations were performed using custom-developed algorithms in MATLAB (release 2024a, The MathWorks, Inc., Natick, MA, USA). Theoretical basis of the fractal methods can be found in works by Grassberger [18] and Ott et al. [19], and those for MSTFD can be found in works by Kozma et al. [20] and Martinez et al. [21]. Brief explanations on the formulae utilized in the analyses can be found in our previous work [16,17]. The differences in fractal measures between pre-RT and post-RT were also calculated (e.g., ΔBoxFD = Post-RT BoxFD − Pre-RT BoxFD) for utilization to the prognosis of RP occurrence.

### 2.4. Statistical Analyses and Feature Selection

Statistical analyses were performed using R statistical software version 4.5.1 (R Foun-dation for Statistical Computing, Vienna, Austria). Wilcoxon signed-rank tests were used to compare pre-RT and post-RT fractal dimensions, given the paired nature of the data and potential non-normality. Descriptive statistics were used to summarize patient characteristics and feature distributions.

The occurrence of RP of grade ≥2 was the primary endpoint of this study. A comprehensive set of features was considered for the predictive model, including patient demographics (age, sex), smoking history, pre-RT chest CT findings (presence of underlying lung disease), radiotherapy planning parameters (mean lung dose, MLD; percentage volume of lung receiving ≥ 5 Gy, V5; ≥10 Gy, V10; and ≥20 Gy, V20), pre-RT fractal dimensions (BoxFD, Lacunarity, MSTFD), and the differences in fractal measures between pre- and post-RT (ΔBoxFD, ΔLacunarity, ΔMSTFD).

Subsequently, we built a random forest model (RFM) to predict RP ≥ grade 2 occurrence and to deduce important features. Using the three features with the highest importance, decision tree model was built for both the RT alone group and the CCRT group, and its prediction performances were assessed. The RFM was trained using a subset of data, and its performance was evaluated using a 5-fold cross-validation method to prevent overfitting. Feature importances was assessed using the Gini impurity reduction method, identifying the most influential predictors of RP.

Based on the most important features identified by the RFM, simplified decision tree models were developed. This decision tree aimed to provide a clinically interpretable and actionable framework for RP risk stratification. The performances of the decision tree models were evaluated using standard metrics: accuracy, Area Under the Receiver Operating Characteristic curve (AUROC), and F1 score. *p*-values were also calculated to test the hypothesis that the accuracy of the decision tree is greater than the No-Information Rate (NIR), providing statistical validation of its predictive power.

## 3. Results

### 3.1. Baseline Characteristics and RP Incidences

The study comprising two cohorts: RT alone group with 85 patients and CCRT group with 81 patients, all diagnosed with NSCLC. Baseline characteristics, including age, sex, smoking status, and radiotherapy planning parameters, as well as the distribution of RP grades observed in this cohort, are summarized in Table 1. RP of grade ≥ 2 occurred in 19 patients (22.3%) treated with RT alone and in 44 patients (54.3%) who received CCRT.

### 3.2. Changes in Fractal Dimensions Post-Radiotherapy

Lacunarity of the lung parenchyma showed distinct changes following radiotherapy in both RT alone and CCRT groups. In RT alone group, lacunarity increase in post-RT comparted to pre-RT, with a median difference of 0.009 (interquartile range (IQR): −0.012–0.026, *p* = 0.024) as determined by the Wilcoxon signed-rank test. In CCRT group, lacunarity also increased by 0.016 (IQR: −0.006–0.040, *p* < 0.001). This suggests that there were increases in the degree of heterogeneity and spatial gaps within the lung tissue followed by RT. In contrast, BoxFD and MSTFD did not show a statistically significant difference between pre-RT and post-RT measurements (all *p*-values > 0.9, see Table 2). This suggests that while the heterogeneity of the spatial distribution of the lung tissue increased, the space filling property and fibrous connectivity remained relatively stable or exhibited non-significant changes in response to radiation. The specific values for pre-RT and post-RT BoxFD, Lacunarity, and MSTFD, along with their standard deviations, are presented in Table 2. A representative case’s pre- and post- RT CT scan and their corresponding NAA masks are shown in Supplementary Figure S1.

### 3.3. Feature Importance in Random Forest Model

The Random Forest Models (RFM) were trained to identify the most important features for predicting RP of grade ≥ 2. For the RT alone group, the RFM feature importance analysis revealed that pre-RT MSTFD and lung V10 values were of the highest importance. For the CCRT group, the most important features were lung V20, ΔBoxFD, Δlacunarity, lung V5, and pre-RT MSTFD. These findings highlight the combined prognostic power of traditional dosimetric parameters and novel fractal imaging biomarkers. Other features, such as age, sex, smoking history also contributed to the model but with lower importance scores. The importance scores can be found in Supplementary Figure S2.

### 3.4. Decision Tree Model Performance for RP Prediction

**Figure 1:**
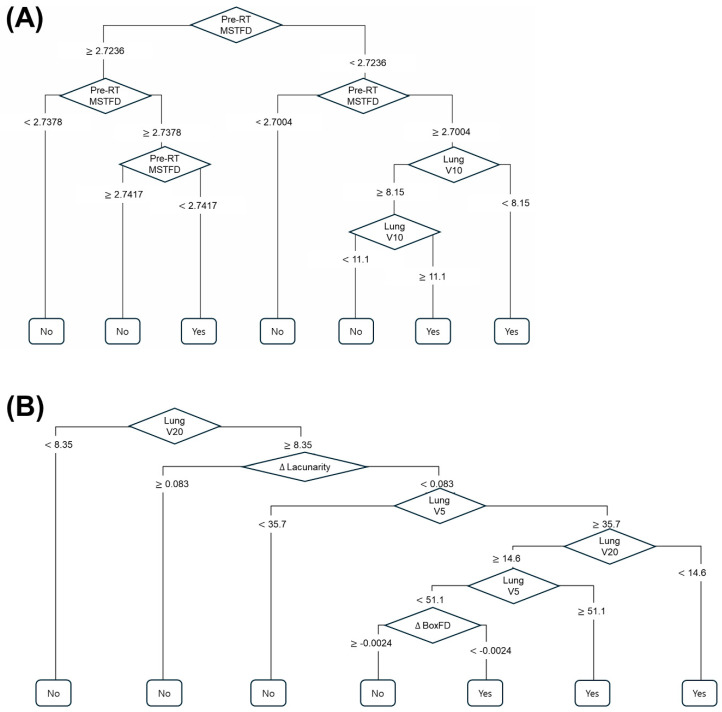
Decision tree models for prediction of radiation pneumonitis ≥ grade 2 in patients receiving definitive radiotherapy alone (**A**) and in patients undergoing concurrent chemoradiotherapy (**B**). Pre-RT MSTFD, pre-radiotherapy minimum spanning tree fractal dimension; Lung V5/10/20, percentage volume of lung receiving ≥ 5/10/20 Gy; Δ Lacunarity, difference in lacunarity between pre- and post- radiotherapy; Δ BoxFD, difference in box-counting fractal dimension between pre- and post-radiotherapy.

Based on the selected features, decision tree models were constructed for the prediction of RP of grade ≥ 2. The structure of the decision tree, including the splitting criteria and terminal nodes, is visually represented in Figure 1. The pre-RT MSTFD feature was incorporated into the initial construction of the decision tree model for CCRT subgroup, but the specific branch that used pre-RT MSTFD was removed during the pruning process to prevent over-fitting. In the RT alone group, the decision tree model demonstrated predictive performances of: accuracy 0.87; AUROC 0.83; F1 Score 0.92. In the CCRT group, accuracy 0.80; AUROC 0.85; F1 score 0.76, respectively. The confusion matrices of the decision tree models are also in Supplementary Tables S1 and S2.

Furthermore, *p*-values less than 0.05 were obtained for the hypothesis that the accuracy of the decision tree is greater than the No-Information Rate (NIR). The *p*-values were 0.021 in the RT alone group and less than 0.001 in the CCRT group. This statistically significant *p*-value strongly supports the model’s ability to predict RP of grade ≥ 2 better than random chance. The decision tree provides a clear, interpretable pathway for risk stratification, allowing clinicians to identify patients at higher risk of developing severe RP based on readily available clinical and imaging parameters.

## 4. Discussion

RP represents a clinically significant late complication that can adversely affect treatment outcomes in patients with lung cancer. Despite its clinical relevance, reliable and widely applicable prediction models remain limited. In the present study, we developed predictive models by integrating fractal- and radiomics-derived imaging biomarkers (BoxFD, lacunarity, MSTFD) with clinical and dosimetric parameters (mean lung dose, V5, V10, V20), employing Random Forest and Decision Tree algorithms. The models demonstrated favorable discriminative performance (AUROC 0.83–0.85, F1 up to 0.92), supporting their potential utility for early identification of patients at increased risk of RP requiring corticosteroid therapy.

Importantly, a significant post-radiotherapy increase in lacunarity was observed, reflecting radiation-induced structural remodeling of lung parenchyma. This finding suggests that RP is associated with elevated spatial heterogeneity, which is consistent with known pathological processes such as inflammation and fibrosis [22]. In contrast, no significant alterations were observed in BoxFD or MSTFD, indicating that these remodeling processes did not substantially affect overall space-filling properties or fiber connectivity. For instance, in conditions such as emphysema, one would expect both a reduction in space-filling properties and an increase in lacunarity [23], which was not evident in the current cohort.

To the best of our knowledge, this is the first study to characterize changes in morphometric complexity features before and after radiotherapy in the context of RP. These results underscore lacunarity as a potentially sensitive imaging biomarker of radiation-induced tissue heterogeneity, and demonstrate the feasibility of incorporating morphometric complexity metrics with clinical and dosimetric parameters for predictive modeling.

In the RFM, pre-RT MSTFD was identified as one of the most important features along with the total lung V5, V10, or V20 in both the RT alone group and in the CCRT group. The total lung V5, V10, and V20 are the well-known dosimetric predictors of RP [24,25], and its combination with MSTFD features enhanced predictive power. In our previous study, MSTFD in simulation CT proved its prognostic power for RP of grade ≥ 2 occurrence, and the current finding seems to be in line with the fact that morphometric complexity in terms of fiber connectivity better represents lung tissue integrity or susceptibility to radiation damage than BoxFD or lacunarity. The findings offer crucial insights into the intricate mechanisms underlying radiation-induced lung injury and RP.

With the most important morphometric complexity and dosimetric features identified through RFM, we developed a clinically interpretable decision tree model for predicting grade ≥ 2 RP. Our aim was to construct a model that achieved satisfactory performance using parameters readily obtainable in routine radiotherapy practice. The morphometric complexity features require no additional laboratory tests, clinical procedures, specialized equipment, or reagents beyond standard radiotherapy, nor do they pose additional risk to patients. Therefore, this prediction model may be applicable in any clinical setting where radiotherapy is performed. Furthermore, early identification of high-risk patients prior to treatment could enable personalized management strategies, including optimization of lung dose parameters, use of advanced radiation techniques (e.g., IMRT, image- or respiration-guided radiotherapy), more frequent short-term monitoring (e.g., monthly rather than quarterly follow-ups), and timely prophylactic or supportive interventions such as corticosteroids or anti-fibrotic agents.

The decision tree model was built for simplicity and interpretability with some trade-offs in prediction performances. Although the decision tree models were simplified, they showed acceptable performances (accuracy 0.87; AUROC 0.83; F1 Score 0.92 in the RT-alone subgroup, accuracy 0.80; AUROC 0.85; F1 score 0.76 in the CCRT subgroup, respectively). The confusion matrices in the Supplementary Tables S1 and S2 show that the false negatives are more prevalent than false positives in RT alone subgroup, whereas the opposite is found in the CCRT subgroup. False negatives represent underestimation of RP risk, likely occurred in patients whose risk was driven by a subtle interplay of several less-dominant features that were excluded from the simplified decision tree model. Conversely, the false positives indicate that the model over-predicts risks for some patients. However, the interpretation of the decision tree models’ performance metrics should be cautious since they were not externally validated yet, due to the lack of multi-centric data.

This study was conducted as a retrospective, single-institution analysis, which may introduce selection bias. In addition, inclusion was limited to patients with diagnostic CT scans obtained before radiotherapy and within six months after treatment, resulting in a relatively small sample size and constraining the generalizability of our findings. Nevertheless, this study provides preliminary evidence supporting the utility of morphometric complexity features for RP prediction, thereby contributing to the existing body of literature and laying the groundwork for future prospective, multicenter investigations.

To address the limitations of prior retrospective single-institution studies, we are currently establishing a large, multi-institutional prospective cohort. By integrating quantitative imaging biomarkers with clinical and dosimetric data, we aim to develop a more robust and generalizable prediction model for RP risk. Furthermore, we are working toward clinical translation of these findings by developing a patient-tailored decision support platform, including a mobile application for systematic monitoring of post-radiotherapy complications, to enable early identification of high-risk patients and support personalized treatment planning.

## 5. Conclusions

Morphometric complexity imaging biomarkers offer valuable prognostic information for predicting grade ≥ 2 RP. The proposed decision tree models may aid in effective RP risk stratification and hold potential as practical tools for guiding prognosis and clinical decision-making in patients with NSCLC undergoing thoracic radiotherapy.

## Figures and Tables

**Table 1:** Baseline characteristics.

	RT Alone (N = 85)		CCRT (N = 81)	
Characteristics	Number	%	Number	%
Age [years; median (range)]	79 (38–93)		74 (53–93)	
Sex				
Female	29	34.1%	11	13.6%
Male	56	65.9%	70	86.4%
Smoking Status				
Never smoker	44	51.8%	33	40.7%
Current or Ex-smoker	41	48.2%	48	59.3%
Planning Parameters				
Total lung MLD [cGy; median (range)]	423 (88–1239)		1036 (430–1933)	
Total lung V5 [%; median (range)]	20.0 (5.3–59.1)		49.7 (15.5–96.4)	
Total lung V20 [%; median (range)]	5.9 (0.4–21.4)		17.5 (5.0–363.0)	
≥Grade 2 RP	19	22.3%	44	54.3%
≥Grade 3 RP	6	7.1%	16	19.8%

RT alone, radiotherapy alone; CCRT, concurrent chemoradiotherapy; MLD, mean lung dose; VD, percentage volume of lung receiving ≥ D Gy; RP, Radiation Pneumonitis.

**Table 2:** Morphometric complexity biomarkers measured pre- and post- RT and their differences.

	Pre-RT	Post-RT	Difference (Post–Pre)	*p*-Value *
**RT alone (N = 85)**				
BoxFD [median (IQR)]	2.315 (2.266–2.363)	2.297 (2.249–2.332)	−0.015 (−0.055–0.014)	0.988
Lacunarity [median (IQR)]	0.139 (0.119–0.175)	0.149 (0.131–0.181)	0.009 (−0.012–0.026)	0.024
MSTFD [median (IQR)]	2.734 (2.707–2.751)	2.728 (2.701–2.751)	−0.005 (−0.028–0.011)	0.978
**CCRT (N = 81)**				
BoxFD [median (IQR)]	2.349 (2.292–2.383)	2.307 (2.266–2.349)	−0.024 (−0.073–0.006)	0.999
Lacunarity [median (IQR)]	0.137 (0.117–0.156)	0.152 (0.135–0.174)	0.016 (−0.006–0.040)	0.00004
MSTFD [median (IQR)]	2.745 (2.716–2.767)	2.725 (2.694–2.743)	−0.015 (−0.042–0.005)	0.999

* Wilcoxon signed rank test with continuity correction; RT alone, radiotherapy alone; CCRT, concurrent chemoradiotherapy; BoxFD, Box-counting Fractal Dimension; MSTFD, Minimum Spanning Tree Fractal Dimension; Note: The fractal dimensions are real numbers without any unit.

## Data Availability

The datasets used and/or analyzed in the current study can be obtained from the corresponding author upon reasonable request.

## Supplementary Materials

The following supporting information can be downloaded at: https://www.mdpi.com/article/10.3390/life15101596/s1, Figure S1: A representative case’s (A) axial reconstruction of CT scan in pre-radiotherapy and its (B) normal attenuation area, (C) CT scan in post-radiotherapy and its (D) normal attenuation area; Figure S2: Variable importance plot for Random Forest Models in patients treated with RT alone (left) or concurrent chemotherapy (right); Table S1: Confusion matrix of the decision tree model in predicting radiation pneumonitis ≥ grade 2 events in patients receiving definitive radiotherapy alone; Table S2: Confusion matrix of the decision tree model in predicting radiation pneumonitis ≥ grade 2 events in patients undergoing concurrent chemoradiotherapy.

## Abbreviations

RP, radiation pneumonitis; NSCLC, non-small cell lung cancer; CCRT, concurrent chemoradiotherapy; IMRT, intensity-modulated radiation therapy; SABR, stereotactic ablative radiotherapy; IPF, idiopathic pulmonary fibrosis; CT, computed tomography; BoxFD, box-counting fractal dimension; MSTFD, minimum spanning tree fractal dimension; CTCAE, Common Terminology Criteria for Adverse Events; RFM, random forest model; AUROC, Area Under the Receiver Operating Characteristic curve; MLD, mean lung dose; VD, percentage volume of lung receiving ≥ D Gy.
