# Supplementary material for: Radiation Pneumonitis Risk Assessment Using Fractal Analyses in NSCLC Patients Treated with Curative-Intent Radiotherapy
Source: Life (Basel). 2025 Oct 13;15(10):1596. doi: 10.3390/life15101596 (PMC12565569; doi:10.3390/life15101596)
Supplement: Supplementary file 1 [file life-15-01596-s001.zip › life-3883067-supplementary.pdf]

Supplementary Materials

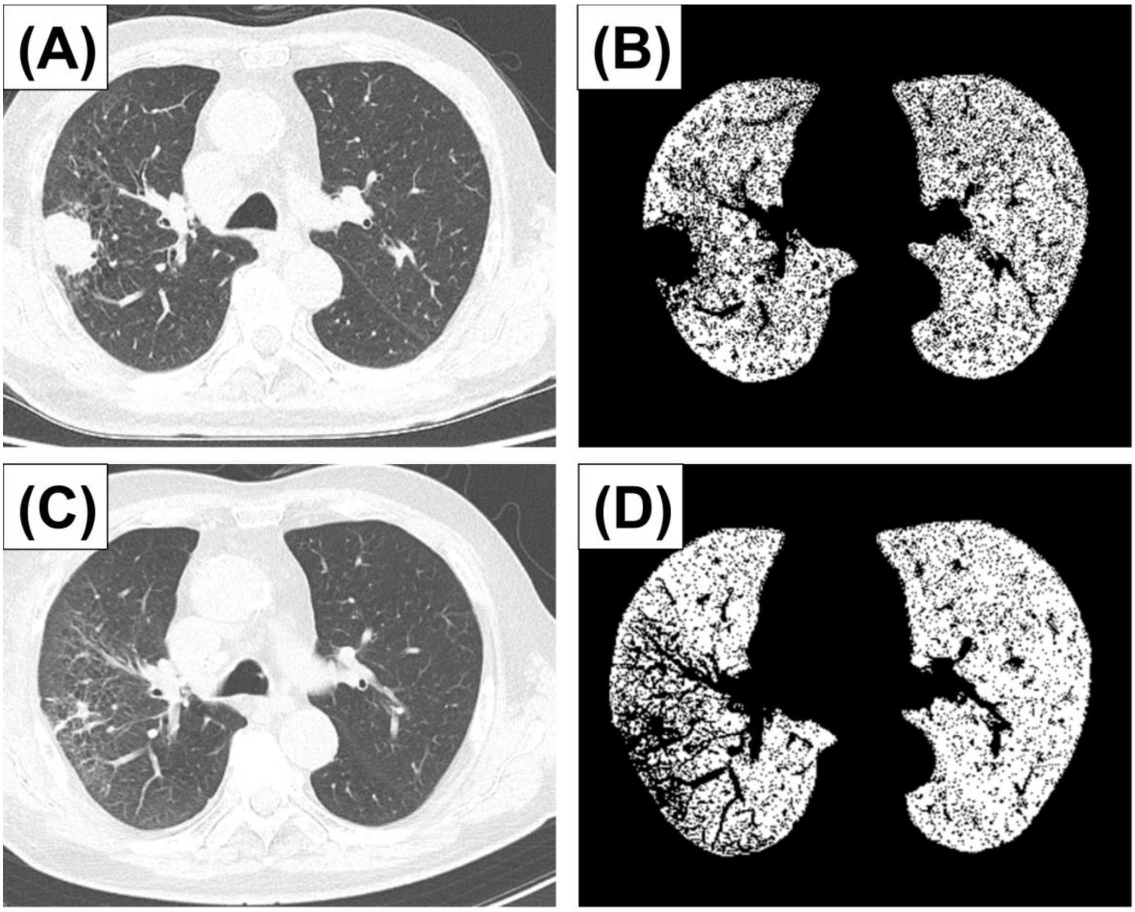

Figure S1. A representative case's (A) axial reconstruction of CT scan in pre-radiotherapy and its (B) normal attenuation area, (C) CT scan in post-radiotherapy and its (D) normal attenuation area.

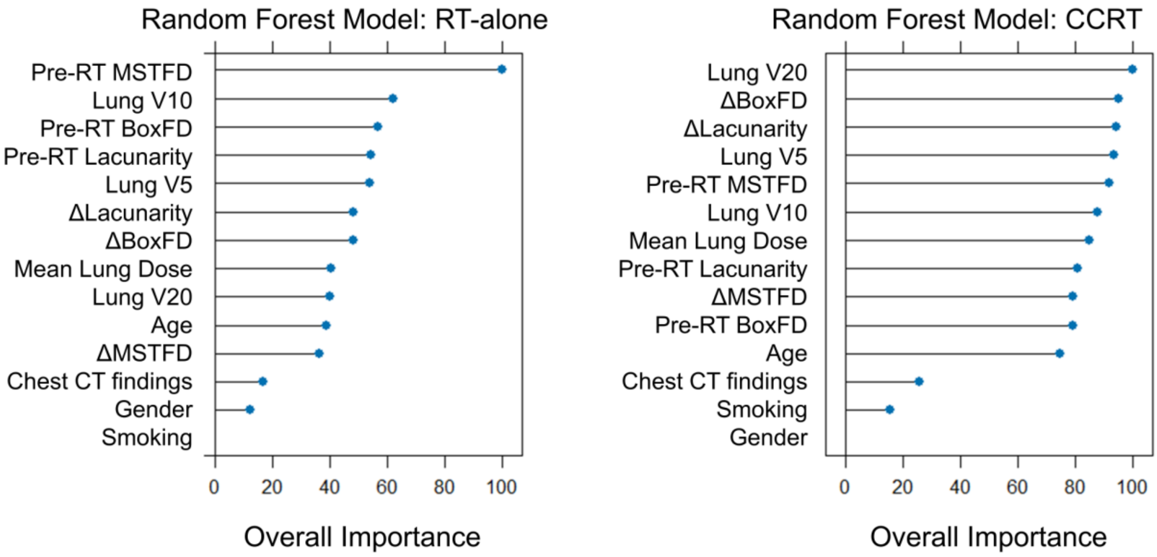

Figure S2. Variable importance plot for Random Forest Models in patients treated with RT alone (left) or concurrent chemotherapy (right).

Table S1. Confusion matrix of the decision tree model in predicting radiation pneumonitis ≥ grade 2 events in patients receiving definitive radiotherapy alone.

	Reference	
Prediction	No	Yes
No	63	8
Yes	3	11

Table S2. Confusion matrix of the decision tree model in predicting radiation pneumonitis ≥ grade 2 events in patients undergoing concurrent chemoradiotherapy.

	Reference	
Prediction	No	Yes
No	25	4
Yes	12	40
